# Earlier Experience of Robotic Inguinal Hernia Repair With the New Hugo™ Robotic System in Europe

**DOI:** 10.3389/jaws.2025.13880

**Published:** 2025-03-31

**Authors:** Valentina Ferri, Emilio Vicente, Yolanda Quijano, Faruk Hernandez, Hipolito Duran, Eduardo Diaz, Isabel Fabra, Luis Malave, Pablo Ruiz, Luca Ballelli, Alessandro Broglio, Camilla Farè, Daniele Cerbo, Alberto Lado, Patricia Hidalgo, Riccardo Caruso

**Affiliations:** ^1^ Hospital Universitario HM Sanchinarro, HM Hospitales, Facultad HM de Ciencias de la Salud de la Universidad Camilo José Cela, Instituto de Investigación Sanitaria HM Hospitales, Madrid, Spain; ^2^ General Surgeon and Gastroenterology Fellow, University of Cartagena, Cartagena, Colombia; ^3^ General Surgical Residency, University of Perugia, Perugia, Italy; ^4^ General Surgical Residency, Univiersity of Pavia, Pavia, Italy; ^5^ General Surgical Residency, University of Rome “La Sapienza”, Roma, Italy

**Keywords:** robotic surgery, minimally invasive surgery, inguinal hernia repair, robotic platform, Hugo™ RAS

## Abstract

**Introduction:**

Minimally invasive robotic surgery has increasingly gained acceptance in abdominal wall surgery. The Hugo™ robotic system, with its modular design, offers enhanced maneuverability and flexibility, making it a promising alternative platform for inguinal hernia treatment. This article aims to present our experience with robotic inguinal hernia repair using the Hugo™ system, focusing on clinical outcomes and the challenges encountered during the learning curve.

**Materials and Methods:**

Since the introduction of the Hugo™ system in our department in January 2023, all patients undergoing robotic inguinal hernia repair with this platform have been prospectively enrolled in this study. Preoperative, intraoperative, and postoperative data were collected and analysed to assess the outcomes.

**Results:**

A total of 69 inguinal hernia repairs were performed using the Hugo™ system in 40 patients, including 29 bilateral and 11 unilateral inguinal hernias. The median console time was 37 min for unilateral hernia while the total procedure time was 45 min (range 30–70 min). The median console time was 94 min for bilateral hernia while the total procedure time was 121.1 min (range 65–236 min). The median docking time for the robotic system was 9.5 min (range: 4.8–20.1 min). No intraoperative complications were observed and only postoperative hematoma was identified and treated.

**Conclusion:**

Robotic inguinal hernia repair with the Hugo™ system is a safe, reproducible, and effective procedure. For teams with a strong background in robotic surgery, the learning curve with the Hugo™ system is rapid, allowing for efficient adaptation of the system to the existing workflow.

## Introduction

Abdominal wall surgery has seen a significant transformation in recent years. What was once a traditional practice has now evolved into a cutting-edge discipline, driven by advancements in minimally invasive techniques. These innovations have illuminated the complex anatomy of the abdominal wall, enabling the development of increasingly sophisticated surgical methods. Within this dynamic landscape, robotic surgery has emerged as a natural progression, addressing the limitations of laparoscopic approaches while enhancing intraoperative outcomes [1].

Since the introduction of robotic technology in surgery, the da Vinci surgical system (Intuitive Surgical, Sunnyvale, CA, United States) has been the most prominent [2]. The evolution of robotic platforms has focused on overcoming limitations in arm maneuverability. Earlier systems, like the da Vinci Xi, featured arms extending from a single pivot point, which restricted their range of motion [3]. Modular systems such as Senhance and Versius were subsequently developed to address these limitations [4, 5].

Medtronic introduced the Hugo™ Robotic Assisted Surgery (RAS) system in 2019; it is a modular robotic-assisted surgery platform designed to improve maneuverability and docking angles. It received approval for urological and gynecological procedures initially, such as radical prostatectomy and total hysterectomy. In November 2022, it received approval for general surgery procedures as well. The Hugo™ RAS system features an open console, a system tower, and four independent arm carts. The surgeon’s head is positioned in front of a large HD 3D monitor, providing a clear view of the patient, robotic arms, and surgical staff. The console includes hand controllers with double function triggers, foot pedals for instrument control, and a head tracking system [6]. This robotic system perfectly meets the needs of abdominal wall surgery in general, and inguinal hernia surgery in particular.

This study aims to report the largest series of patients undergoing inguinal hernia repair with the new Hugo™ robotic system, providing insights into intra and postoperative outcomes and learning curve.

## Materials and Methods

This is a prospective study. Since January 2023, all patients scheduled for robotic unilateral or bilateral inguinal hernia repair were enrolled. Age over 18 years, unilateral and bilateral inguinal hernia and patients accepting to undergo robotic surgery were the inclusion criteria. Inguinal and inguinoscrotal hernias were classified according to the EHS society classification [6, 7].

### Hugo™ RAS System

Hugo™ RAS system has joined our team with extensive experience using the Da Vinci system, which has performed over 500 procedures previously. Since the first surgery performed with Hugo™ at our center in January 2023, we have completed 75 surgeries using this platform, most of which involved the abdominal wall and benign pathologies of the esophagus and gallbladder.

### Surgical Technique

#### Trocar Placement

**FIGURE 1:**
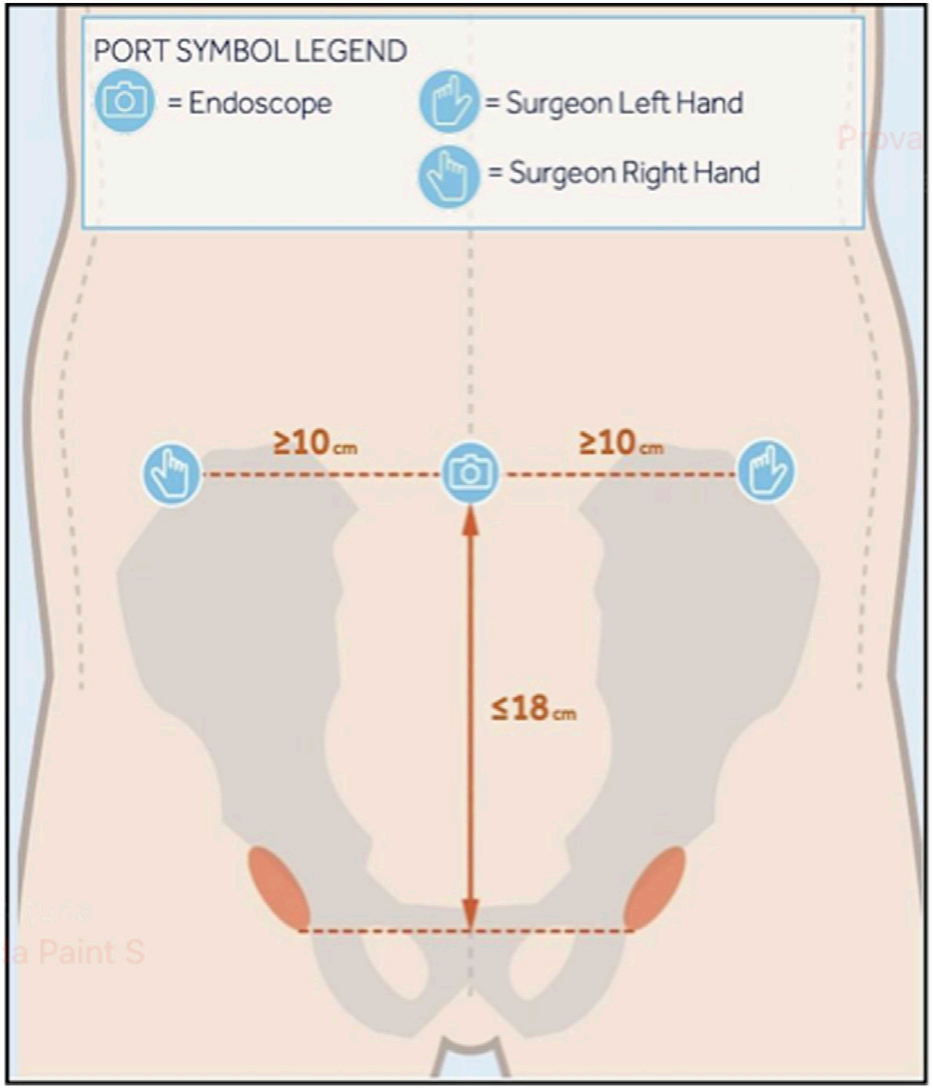
Set up guide for port placement.

**FIGURE 2:**
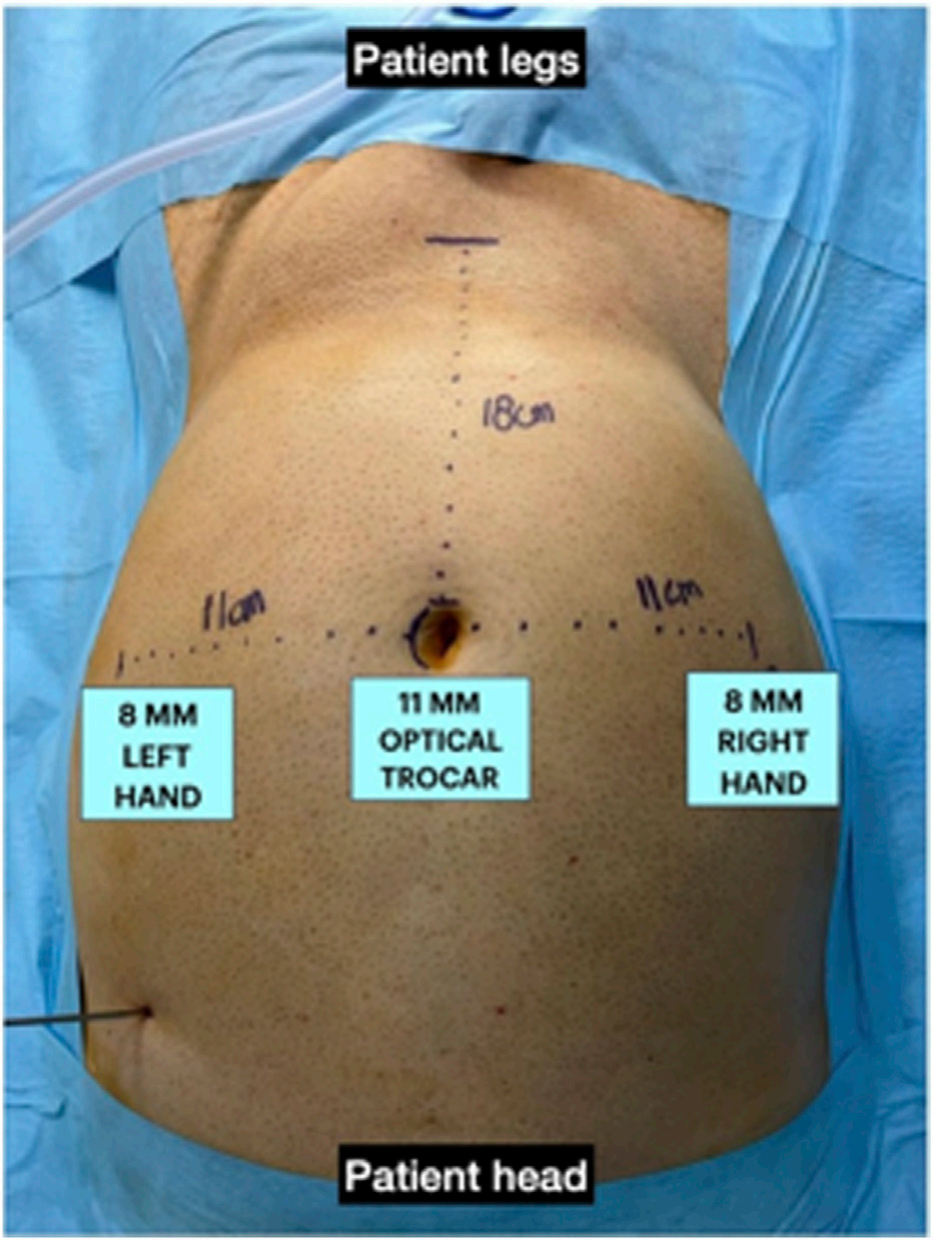
Port placement.

A pneumoperitoneum was created at 14 mmHg using CO2 gas insufflation delivered through a Veress needle, then an 11 mm trocar is inserted just above the umbilicus. A 30-degree optic is introduced through the trocar for the exploration of the abdominal cavity, and the remaining two trocars have an 8 mm size on the transverse umbilical line. The endoscope and surgeon’s hand ports are placed no more than 18 cm above the pubic bone on the transverse umbilical line, and at least 2 cm away from all body ports and prominences (Figures 1, 2).

#### Docking

**FIGURE 3:**
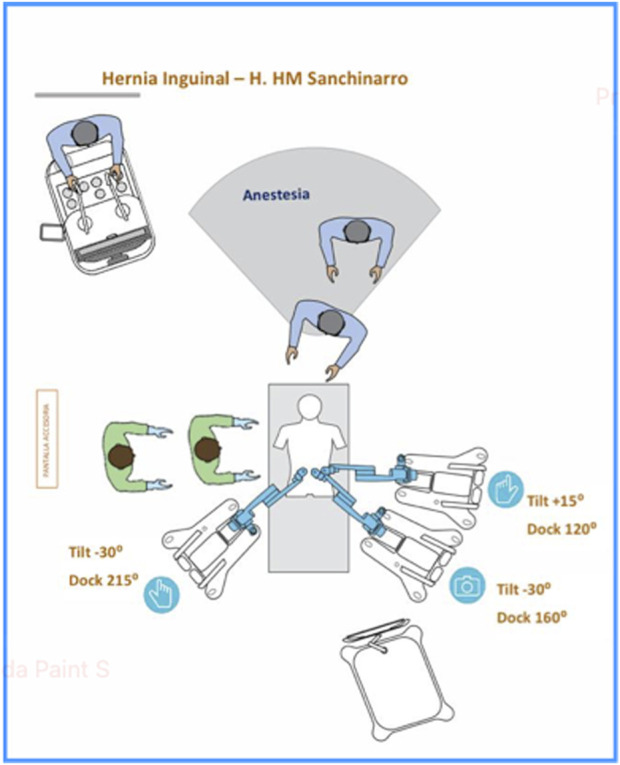
Set up guide of docking and tilt angles configuration.

The patient is placed in a Trendelenburg position of more than 15°, with legs closed, ensuring that all port incisions are at the correct height. For docking, two robotic arms were positioned on the patient’s left side for the optical and left trocars, while one arm was placed on the right side. The angles of tilt and docking are illustrated in Figure 3.

#### Arm Configurations

Arm 1: Tilt angle of +15°, docking angle of 120°. Arm 2: Tilt angle of −30°, docking angle of 160°. Arm 3: Tilt angle of −30°, docking angle of 215°.

**FIGURE 4:**
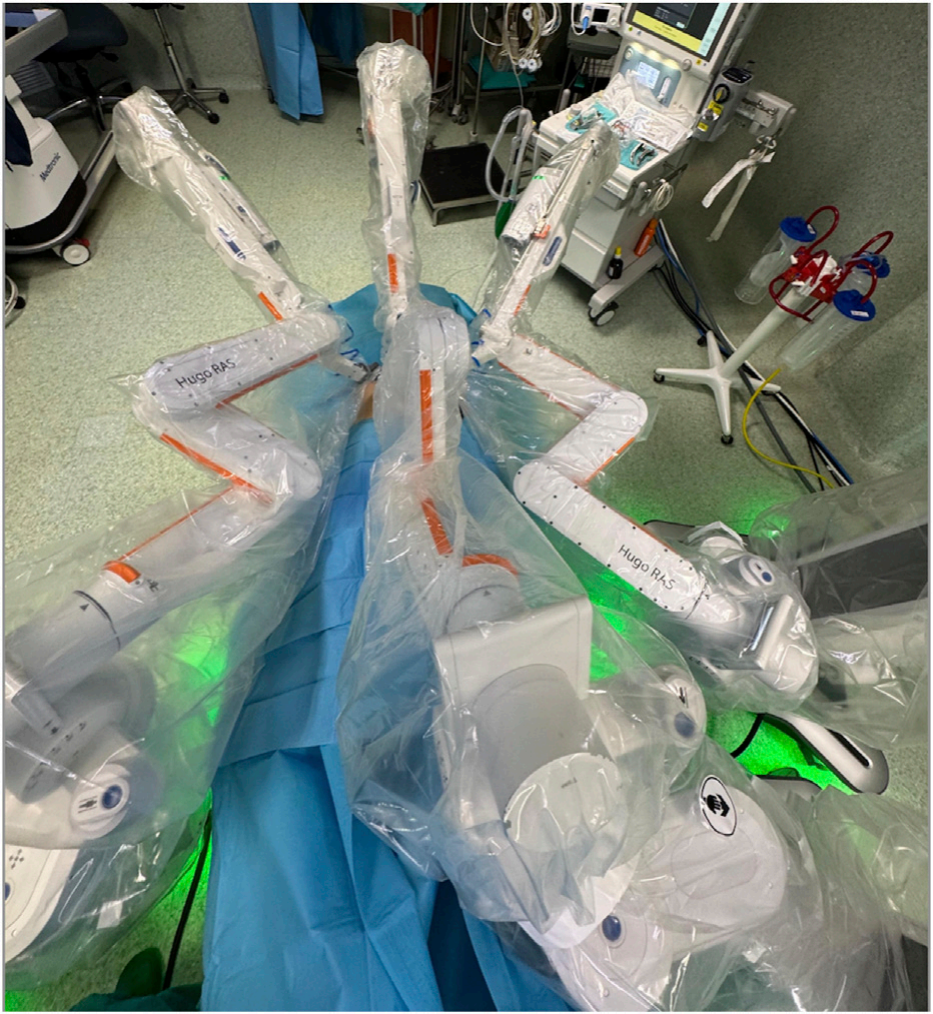
Final configuration of surgical theatre.

**FIGURE 5:**
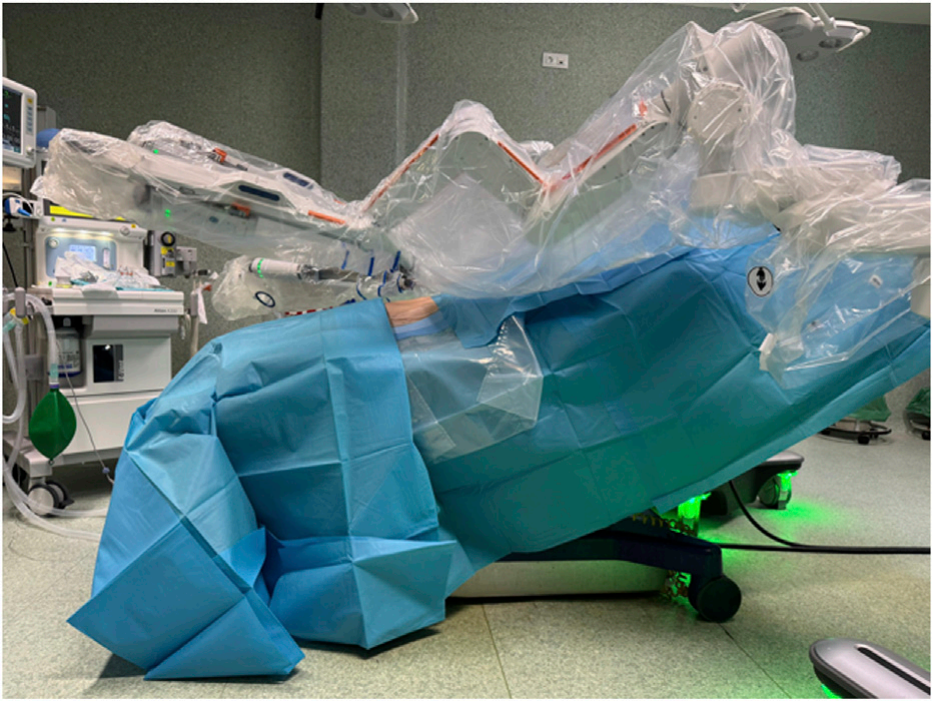
Final configuration of surgical theatre, lateral view.

Despite the previously mentioned standards for patients-specific port placement, based on our experience, we have proposed a modification of arm placement for inguinal hernia repair. This modification was made in accordance with the Medtronic team as an alternative to the standard configuration: we have adjusted the arm height from 70 cm to 80 cm, and the tilt angle of the operating table has been raised to more than 15° in the Trendelenburg position. These modifications enhance port maneuverability, preventing collisions and ensuring that the workstations do not obstruct the patient’s head (Figures 4, 5).

#### Surgical Steps

The initial step involves creating the peritoneal flap, then the procedure is performed in the same way as laparoscopic TAPP [8]. The hernia is repaired using an ENDOLAP 3D mesh (produced by the DynaMesh^®^ Company) of 15 × 10 cm, which was secured with Histoacryl^®^ glue (Braun Surgical Gmbh, Melsungen, Germany). Intracorporeal suturing of peritoneal flap was performed.

### Data Analysis

Docking time was determined by the time since the first arm was initiated. The cart was rotated toward the patient until all arms were connected to the ports and the robotic instruments were inserted into the abdomen. Console time was determined as the time from docking to undocking. The total operative time was determined as the time from the skin incision to the end of the skin suture.

## Results

A total of 69 inguinal hernia repairs were performed using the Hugo™ system across 40 patients, which included 29 bilateral and 11 unilateral inguinal hernias. The median age of the patients was 65.4 years (range: 40–86 years), with 37 male patients (92%) and 3 female patients (8%). The ASA scores were distributed as follows: 14 patients (35%) had a score of I, 15 patients (37.5%) had a score of II, and 11 patients (27.5%) had a score of III (range: I–III). The median BMI was 26.3 kg/m2 (range: 22–33.2 kg/m2) (see Table 1).

**TABLE 1:** Demographic preoperative data.

	Unilateral Hernia	Bilateral Hernia	Total Patients
Number of Patients	11	29	40
Sex (F/M)	1 (9%)/10 (91%)	2 (7%)/27 (93%)	3 (7.5%)/37 (92.5%)
Age (years, median, range)	63.1 (40–79)	66.3 (42–86)	65.4 (40–86)
ASA Score (n %)			
I	4 (36%)	10 (34%)	14 (35%)
II	4 (36%)	11 (38%)	15 (37%)
III	3 (28%)	8 (28%)	11 (28%)
BMI (kg/m², median, range)	25.4 (22–33.2)	26 (24–32.1)	26.3 (22–33.2)
Hernia Classification (n %)			
L1-2	9 (81%)	21 (72%)	30 (75%)
Inguino-scrotal	2 (19%)	8 (28%)	10 (25%)
Recurrent Hernia			
Yes	5 (45%)	6 (21%)	11 (27.5%)
No	6 (55%)	23 (79%)	29 (72.5%)

**FIGURE 6:**
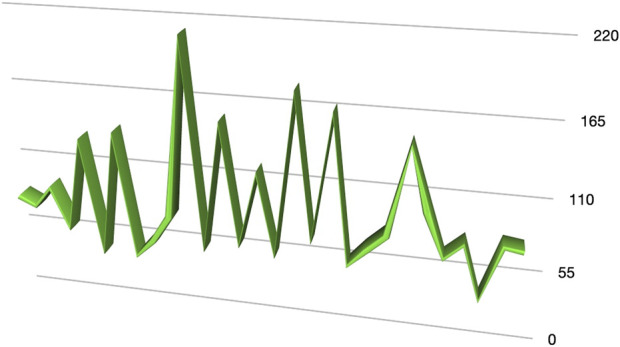
Console time.

**FIGURE 7:**
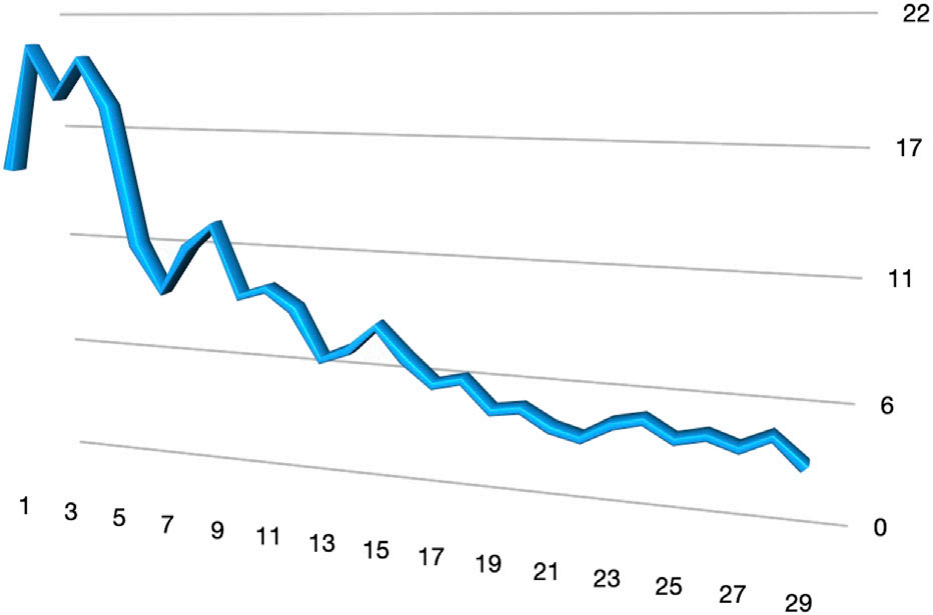
Docking time.

Among the cases, 10 patients (25%) presented with inguinoscrotal hernias, 7 patientes presented an S1 and 3 patients presented an S2 inguinoscrotal hernia according to the EHS Classification [6, 7]. For unilateral hernia the median console time was 37 min (20–55 min) while the total procedure time was 45 min (range 30–70 min). For bilateral hernia the median console time was 94 min (range 54–214 min) while the total procedure time was 121.1 min (range 65–236 min). The median docking time for the robotic system was 9,5 min (range: 4.8–20.1 min). In Figures 6, 7, the variation in console time and docking time across the different procedures performed can be observed. After 10 procedures the docking time decreases from a median of 9,5 min to 6,7 min, while we do not observe variation in the console time. No intraoperative complications or conversions to open surgery were observed. The median hospital stay was 1.3 days (range: 1–3 days) (Table 2). During postoperative follow-up, an older patient developed an inguinal hematoma. This patient, who had an inguinoscrotal hernia and was receiving anticoagulation therapy for atrial fibrillation and was successfully treated with radiologically guided needle drainage.

**TABLE 2:** Intraoperative and postoperative data.

	Unilateral Hernia (n = 11)	Bilateral Hernia (n = 29)	Total (n = 40)
Docking Time, median (range) (minutes)	-	-	9.5 (4.8–20.1)
Operative Time, median (range) (minutes)	52 (35–61)	113 (75–240)	79.5 (35–240)
Hospital Stay, median (range) (days)	1 (1–1)	1.5 (1–3)	1.3 (1–3)
Postoperative Complications n (%)			
Seroma	0 (0%)	1 (3.5%)	1 (2.5%)
Hematoma	1 (9%)	2 (7%)	3 (7.5%)
Wound Infection	0 (0%)	0 (0%)	0 (0%)
Dindo-Clavien Grade > III n (%)			
Yes	0 (0%)	1 (3.5%)	1 (2.5%)
No	11 (100%)	28 (96.5%)	39 (97.5%)

## Discussion

The field of abdominal wall surgery has undergone a significant transformation in recent years, driven primarily by the advent of minimally invasive techniques. These advancements have provided a deeper understanding of the complex anatomy of the abdominal wall, paving the way for more refined surgical procedures. In this context, robotic surgery has emerged as a crucial innovation, representing the next step in the evolution of laparoscopic surgery with its potential to enhance intraoperative precision and outcomes [9, 10]. Historically, the da Vinci surgical system has dominated the landscape of robotic surgery since its introduction. However, the evolution of robotic platforms has been marked by ongoing efforts to overcome limitations, particularly in terms of the maneuverability of robotic arms [11]. The introduction of the Hugo™ RAS system in 2019 marked a significant advancement in robotic-assisted surgery. Designed with a modular architecture, the Hugo™ RAS system enhances maneuverability and docking precision, offering a more versatile approach to various surgical procedures [12, 13]. Initially approved for urological and gynecological surgeries, such as radical prostatectomy and total hysterectomy, the system received approval for general surgery in November 2022, expanding its applicability [14].

This study represents the largest series of robotic inguinal hernia repairs performed with the Hugo™. The feasibility of robotic-assisted inguinal hernia repair using this platform was clearly demonstrated in this study. Despite the complexity of robotic surgery and the initial learning curve associated with a new surgical platform, our results show that the procedure can be performed efficiently and safely across a variety of cases, including inguinoscrotal hernias. The progressive reduction in docking time, particularly after the first 10 cases, highlights the system’s adaptability and the ease with which surgical teams can become proficient. During our learning curve, the assistants at the operating table have been always the same nurse, who underwent training together with the surgeon. The median console time for unilateral hernia repair was 37 min, and for bilateral cases, it was 94 min. These times are comparable to those reported in other studies utilising robotic platforms, where console times ranged from 30 to 120 min [15, 16]. This suggests that no significant learning curve is required for surgeons with extensive experience in minimally invasive hernia repair when transitioning from laparoscopic or Da Vinci to the Hugo™ RAS system. Initially, docking took a median of 9.5 min, with a significant reduction to 6.7 min after the first 10 procedures. Several modifications to the standard setup of the Hugo™ system were introduced in collaboration with the Medtronic team: the arm height has been increased from 70 cm to 80 cm, and the tilt angle of the operating table has been raised to over 15° in the Trendelenburg position. This configuration reduces potential conflicts between the robotic arms and between the arms and the patient’s head.

In our study, no intraoperative complications or conversions to open surgery were observed, reinforcing the safety profile of robotic-assisted repair. These findings are similar to other reported in other studies on robotic hernia repair, which report low complication rates and no need for conversion to open surgery, even in complex cases [17].

### Final Considerations on Robotic Platforms in Hernia Surgery

Our team has extensive experience in robotic surgery, which began in 2010 with the first model of the da Vinci system. Over the years, we have used three different da Vinci robotic platforms, including the latest Xi model. The Hugo™ platform was introduced in 2022, after more than 500 procedures performed using the da Vinci system. Comparing the two platforms, the modular design of the Hugo™ system offers enhanced flexibility in docking and positioning, allowing for better adaptation to diverse surgical scenarios. However, due to its modular setup, the system occupies more space in the operating room. For this reason, when it is possible, we optimize its use by utilizing three robotic arms instead of the standard four, which reduces spatial constraints while maintaining efficiency. Additionally, the system allows the resolution of conflicts by enabling modifications to the articulation angles of the robotic arms, providing superior adaptability during complex procedures. However, the da Vinci platform provides a more standardized workflow, which can be advantageous for teams less familiar with robotic-assisted surgery.

Both systems provide high-definition three-dimensional visualization. The Hugo™ system is equipped with an open console that offers three-dimensional visualization through the use of specialized glasses, ensuring optimal depth perception during surgery and surgical theatre control.

On the other hand the main limitation of the Hugo™ system is the energy, in fact, it is limited to monopolar and bipolar energy modalities and lacks mechanical staplers and devices for applying hemolock clips, which are available on the da Vinci platform. These limitations may influence the choice of platform based on the complexity of the procedure and the specific tools required.

Nevertheless in our opinion the Hugo™ platform fulfills the essential requirements for hernia surgery and abdominal wall procedures in general. It uses the same energy modalities as laparoscopic surgery, ensuring compatibility with established workflows while providing advanced capabilities. The platform significantly enhances the dissection of abdominal wall planes, improving surgical precision and visibility during the procedure. One of its key benefits is its ability to simplify challenging tasks, such as midline closure, which is often difficult in laparoscopic surgery. By offering superior ergonomics and advanced three-dimensional visualization and image maginfication the Hugo™ system facilitates these procedures with greater ease and efficiency, contributing to improved surgical outcomes.

## Conclusion

Robotic-assisted inguinal hernia repair with the Hugo™ system is a safe and effective approach, with notable improvements in docking time after the initial learning curve; the procedure offers a favorable safety profile with minimal complications and short hospital stays. As robotic surgery continues to evolve, its role in inguinal hernia repair may further expand, particularly as more surgeons gain experience and further improvements in operative efficiency are realized.

## Data Availability

The original contributions presented in the study are included in the article/supplementary material, further inquiries can be directed to the corresponding author.
